# Bi-institutional analysis of microbiological spectrum and therapeutic management of parotid abscesses

**DOI:** 10.1186/s13005-024-00438-w

**Published:** 2024-07-12

**Authors:** Marcel Mayer, Julia Esser, Sarah Victoria Walker, Sami Shabli, Axel Lechner, Martin Canis, Jens Peter Klussmann, Lisa Nachtsheim, Philipp Wolber

**Affiliations:** 1 Department of Otorhinolaryngology, Head and Neck Surgery, Medical Faculty, University of Cologne, Cologne, Germany; 2 Institute for Medical Microbiology, Immunology and Hygiene, Medical Faculty, University of Cologne, Cologne, Germany; 3 Institute for Clinical Microbiology and Hospital Hygiene, RKH Regionale Kliniken Holding Und Services GmbH, Ludwigsburg, Germany; 4 Department of Otorhinolaryngology, Head and Neck Surgery, Medical Faculty, University of Munich, Munich, Germany

**Keywords:** Parotid abscess, Sialolithiasis, Tumor, Pathogenicity, Antibiotics, Treatment strategies, Microbiology

## Abstract

**Background:**

A parotid abscess (PA) is a complication of an acute bacterial parotitis with a potentially life-threatening course. To date, data on the diagnosis and therapy of PA is sparse and mostly consists of case reports or case series. Therefore, this study aimed at comprehensively analyzing the microbiological spectrum and the therapeutic management in a bi-institutional setting.

**Methods:**

A retrospective clinical chart review was performed to identify all patients surgically treated for PA at two tertiary care centers in Germany. Data on demographics, clinical management and microbiological data including species identification, pathogenicity, type of antibiotic therapy, adjustment of antibiotics, antibiotic sensitivity testing, and smear test results were extracted. Intervention-related variables and etiology were analyzed for their statistical association with outcome variables.

**Results:**

Overall, 85 patients were included. Most patients (92.9%) underwent surgical incision. Around half of the patients (45.9%) were treated under local anesthesia. No facial nerve palsy was observed. The most frequently detected pathogens were *Streptococci* (*n* = 23), followed by *Staphylococcus aureus* (*n* = 6) including one case of methicillin-resistant *Staphylococcus aureus*. Most patients (68.2%) received an aminopenicillin ± beta-lactamase inhibitor as empiric antibiotic therapy. In 6 cases the antibiotic therapy was modified after receiving the antibiogram. Four patients (5.2%) presented with recurrent PA. Etiology was idiopathic (42.4%), followed by tumorous (12.9%), obstructive, and immunosuppressive (each 11.8%). Patients with a dental focus (*p* = 0.007) had a longer duration of hospitalization.

**Conclusion:**

The results show that the surgical therapy of PA under local anesthesia is safe. A dental examination should routinely be performed to rule out a dental focus. Obtaining a microbiological specimen in order to modify antibiotic therapy if necessary and a histopathological specimen to rule out a tumorous etiology is obligate.

## Background

Acute bacterial parotitis accounts for approximately 1.5 out of 10,000 hospital admissions [1]. One of the most relevant and serious complications of acute bacterial parotitis is a parotid abscess (PA). Mainly due to the close anatomical relationship between the parotid gland and the facial nerve, a PA represents a therapeutic challenge in clinical practice. A PA is characterized by a localized collection of pus within the parotid gland, resulting from acute purulent sialadenitis, generally caused by ascending infection via Stensen’s duct or bacteremia [2]. Prompt diagnosis and appropriate management are crucial to prevent complications such as septicemia, osteomyelitis, mediastinitis or facial nerve palsy [3–5].

Previously identified bacterial pathogens are *Staphylococcus aureus*, *Streptococcus spp*., and *Haemophilus influenza* [6]. Other bacteria, including *Escherichia coli*, *Klebsiella pneumoniae*, and anaerobic organisms, have been identified in rare cases [7, 8]. Known risk factors for PA include poor oral hygiene, immune deficiency, sialolithiasis, ductal obstruction, dehydration, and malnutrition [7]. Therapeutic management consists of systemic antibiotics and interventional procedures like surgical incision and drainage or ultrasound-guided needle aspiration [9]. Interventional therapy can be performed under local or general anesthesia depending on patient-specific risk factors (e.g., advanced age, multimorbidity) and the hospital’s standard protocol.

Although there are several studies describing the therapy of PA, most of these are case reports or series [10] resulting in insufficient evidence on the correct treatment and analysis of microbiological data. It is worth noting that a recent systematic review was conducted [10]. However, due to the limited size of case series and the heterogeneous nature of the data, a meta-analysis was not feasible. Moreover, the scarcity of available data has prevented thorough statistical analyses regarding influential factors and outcome variables [10]. Additionally, there is a dearth of comprehensive studies on the etiology of parotid abscesses, mainly due to the small size of previous series. Consequently, the systematic review refrains from drawing conclusions on this matter [10].

Therefore, this bi-institutional study aimed at comprehensively elucidating the etiology, the microbiological spectrum, and treatment strategies of PA to improve patient outcomes and clinical decision-making in the management of PA.

## Methods

All patients diagnosed with PA at the departments of otolaryngology/head and neck surgery of the university hospital Cologne and the university hospital Munich between January 2012 and April 2022 were included in this retrospective study. Diagnosis has been made based on clinical findings and ultrasound in all cases. If necessary for preoperative planning, a CT scan of the head and neck has been performed, additionally. Incision and drainage with drain insertion and antibiotic treatment was the standard of care in both institutions. Ultrasound-guided needle aspiration was performed in case of an abscess with a maximum diameter of 1.0 cm and no sonographic proof of residual pus after aspiration. Drain placement was omitted in case of ultrasound-guided needle aspiration or as individual surgeon’s decision in a limited number of cases. Data on demographics, microbiological aspects and clinical management were collected from the medical records. Patient demographics included sex, age, predisposing factors, and comorbid diseases. Microbiological factors consisted of species identification, pathogenicity, type of antibiotic therapy, adjustment of antibiotics, antibiotic sensitivity testing, and smear test results. Intraoperatively obtained clinical specimen were incubated for 7 days according to standard laboratory protocols. Cultural growth was identified to species level by MALDI-TOF MS (Bruker Daltonic GmbH, Bremen, Germany). Phenotypical resistance was primarily determined by Vitek2® (bioMérieux, Nürtingen, Germany) or, if not successful, by agar disc diffusion each in accordance with EUCAST standards. Results were categorized in dependance of the pathogenicity of the detected species and their combination within the specimen in “highly relevant”,”possibly relevant”, and”probably not relevant”. “Highly relevant” considered were species of the *Streptococcus anginosus* group, *Haemophilus influenzae*, *Staphylococcus* *aureus*, *Francisella* *tularensis* and multiple strict anaerobe species. Polymicrobial specimen containing only physiological oral flora were categorized as “possibly relevant”. Specimen with only 1–2 species of the physiological oral flora of no or low pathogenicity were categorized as "probably not relevant". Species of the oral flora may contribute to PA of course but mainly as co-contributors along with species of higher pathogenicity e.g., Staphylococcus aureus. If solemnly detected without additional pathogenic species the relevance remains questionable and was therefore categorized as "probably not relevant". Data on clinical management included the length of hospital stay, duration of antibiotic administration as well as (surgical) drainage techniques, recurrence rates and complications.

The study was conducted in accordance with the Declaration of Helsinki and approved by the Ethics Committee of the University of Cologne (approval code: 23–1252).

Statistical analyses were performed using IBM SPSS Statistics for Mac version 29.0.1.0 (171) (IBM, Armonk, NY). Categorical data were presented as percentages, while continuous data were expressed as means. The t-test was used to compare continuous variables, and the chi-squared test was employed to assess the relationship between categorical variables. *P*-values lower than 0.05 were considered statistically significant.

## Results

### Patients’ characteristics

A total of 85 patients with PA were included of whom 42 (49.4%) were female. Mean age was 57.5 years (± 21.2), ranging from 1 to 94 years (Table 1). Among the examined cases, 44 (51.8%) PA were located on the right side. Baseline characteristics did not significantly differ between the two centers. The average hospital stay was 7.3 days (± 4.7), with a range of 0 to 30 days, differing between the two hospitals (6.2 ± 2.9 days vs. 8.4 ± 5.8 days, *p* = 0.026) and no difference between genders (*p* = 0.493) or age (≥ 50 vs. < 50 years, *p* = 0.092).

**Table 1:** Patients’ characteristics, procedural characteristics, and etiology

Patient characteristics	*n* (%) or mean ± SD
Gender	42 (49.4, female), 43 (50.6, male)
Mean age (years)	57.5 ± 21.2
Location right side	44 (51.8)
Duration of hospitalisation (days)	7.32 ± 4.7
**Type of anaesthesia**	
Local anaesthesia	39 (45.9)
General anaesthesia	46 (54.1)
**Type of procedure**	
Surgical incision	79 (92.9)
Ultrasound-guided needle aspiration	6 (7.1)
Insertion of wound drain	77 (97.5% of surgically treated patients)
Drain duration (days)	3.3 ± 1.7
**Immunosuppression**	12 (14.1)
Diabetes mellitus	7 (8.2)
Immonosuppressants	3 (3.5)
HIV	1 (1.2)
Myelodysplastic syndrome	1 (1.2)

*SD* Standard deviation

### Surgery and complications

**Fig. 1:**
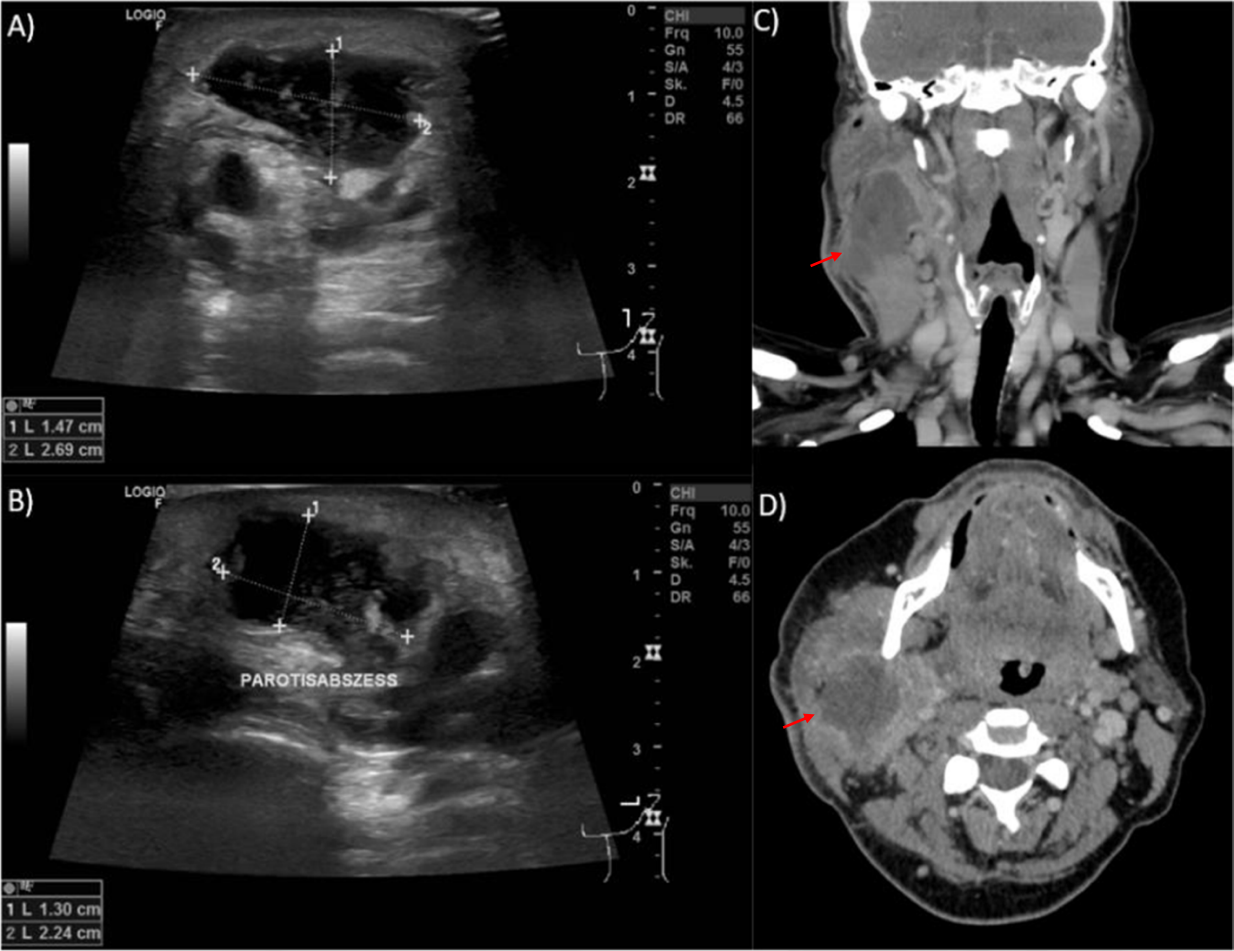
Sonographic and CT imaging of parotid abscesses. **A** Coronal and **B** transverse sonographic images of the left parotid gland displaying a hypoechoic, partly anechoic mass within the parotid gland with partly ill-defined margins and posterior acoustic enhancement and hyperechoic debris in a 60-year-old female patient. **C** Coronal and **D** axial CT scan of the neck with contrast displaying a hypodense partly lobulated mass of the right parotid gland (red arrow) with capsular ring enhancement and surrounding inflammatory changes and contact to the sternocleidomastoid muscle and the mandible in a 51-year-old female patient

The majority of patients underwent surgical incision and drainage (79 cases, 92.9%), while six patients were treated with ultrasound-guided needle aspiration of the PA. Figure 1 displays an exemplary sonography image and a CT scan of patients with PA. Local anesthesia was applied in 39 patients (45.9%), while 46 patients (54.1%) underwent surgery under general anesthesia. There was no significant correlation between type of anesthesia and length of hospital stay (*p* = 0.688). In 77 cases a wound drain was inserted during surgery, which remained for an average of 3.3 days. If a drain was inserted, patients had a significantly longer hospital stay (7.7 days vs. 2.2 days; *p* = 0.005) (Table 2). Out of all patients, nine (10.6%) experienced postoperative complications, with salivary fistula being the most common in five cases (5.9%), followed by residual inflammation, where two patients presented with residual abscesses found in sonography or computed tomography with no further need for surgical intervention (2.4%) (Table 3). There were no occurrences of postoperative facial nerve palsies in either the local anesthesia or general anesthesia groups. Four patients required postoperative parotidectomy after complete resolution of the acute infection due to superinfected adenolymphoma with initial presentation as PA. Four patients (5.2%) experienced recurrent abscesses during the postoperative course, which required revision surgery within 14 days after initial treatment, of whom in one case abscesses reoccurred four times within the first year. This case represented a one-year-old child with a rare abscessed congenital epidermoid cyst of the parotid gland. After first incision and drainage with drain insertion in general anesthesia the cyst showed recurrent abscess formation for four times within one year. The lesion was finally surgically removed during an infection-free interval and the patient remained recurrence-free since then. Another patient suffered from decompensated heart failure during his treatment of PA.

**Table 2:** Statistical association between intervention-related variables and outcome variables

**Variable**	**General anesthesia** (*n* = 46)	**Local anesthesia** (*n* = 39)	***p***
**Duration of antibiotics** in days, mean ± SD	11.43 ± 4.37	10.94 ± 5.06	0.659
**Hospital stay** in days, mean ± SD	7.52 ± 4.49	7.10 ± 5.10	0.688
**Drain duration** in days, mean ± SD	2.84 ± 0.97	3.84 ± 2.36	0.055
**Complications** n (%)	5 (10.9)	4 (10.3)	0.927
**Recurrence** n (%)	3 (6.8)	1 (3.0)	0.459
	**US needle-aspiration** (*n* = 6)	**Surgical incision** (*n* = 79)	***p***
**Duration of antibiotics** in days, mean ± SD	13.16 ± 8.88	11 ± 4.3	0.584
**Hospital stay** in days, mean ± SD	11.5 ± 9.39	7.01 ± 4.15	0.297
**Drain duration** in days, mean ± SD	3.0 ± 1.41	3.31 ± 1.82	0.743
**Complications** n (%)	1 (16.7)	8 (10.1)	0.616
**Recurrence** n (%)	1 (16.7)	3 (4.2)	0.187
	**Drain** (*n* = 77)	**No drain** (*n* = 2)	***p***
**Duration of antibiotics** in days, mean ± SD	11.41 ± 4.74	8.25 ± 2.75	0.192
**Hospital stay** in days, mean ± SD	7.73 ± 4.68	2.17 ± 2.86	**0.005**
**Complications** n (%)	9 (11.5)	0 (0.0)	0.379
**Recurrence** n (%)	3 (4.3)	0 (0.0)	0.605

*SD* Standard deviation, *US needle-aspiration* Ultrasound-guided needle aspiration, significance level < 0.05, significant results in bold letters

**Table 3:** Complications and recurrence

Complications (*n* = 9)	*n *(%)
Salivary fistula	5 (5.9)
Residual inflammation	2 (2.4)
Allergic reaction to antibiotics	1 (1.2)
Heart failure	1 (1.2)
Recurrence of abscess	4 (5.2)

### Etiology and predisposing factors

**Fig. 2:**
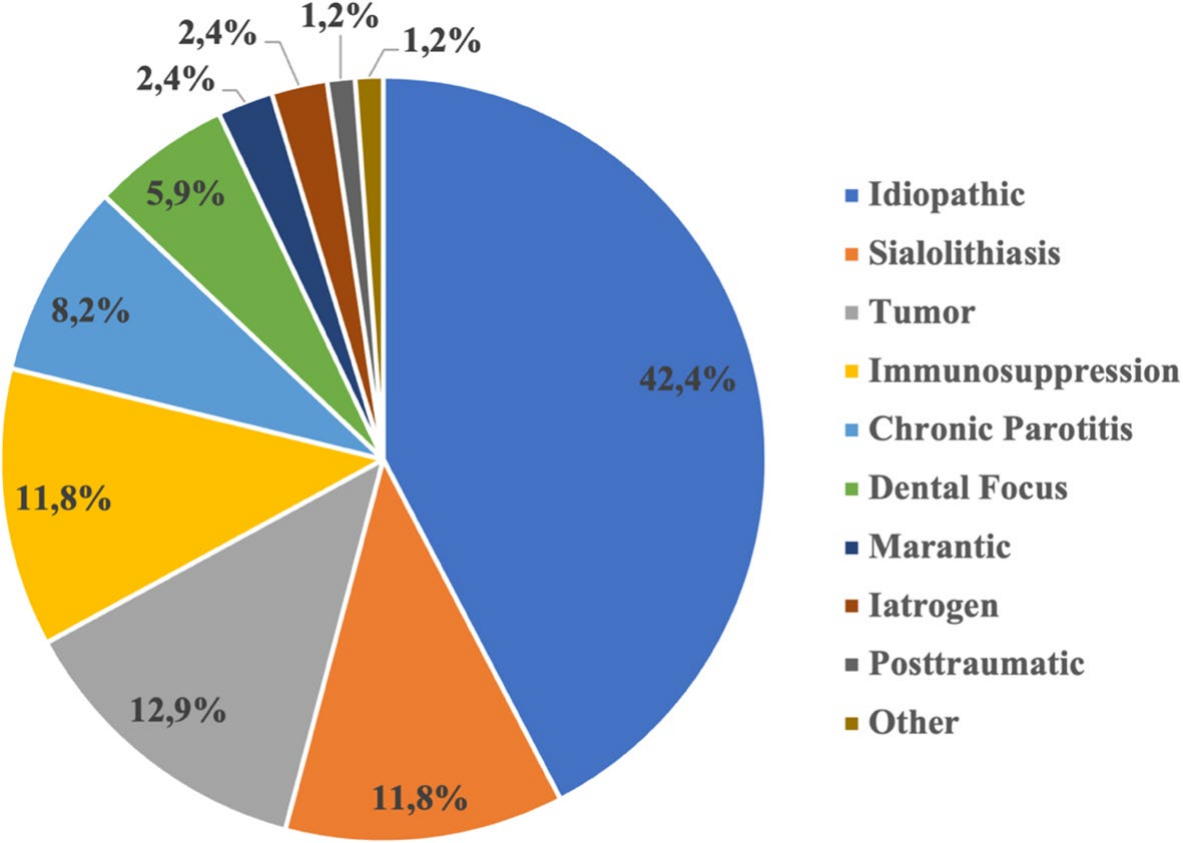
Etiology of parotid abscesses

The majority of abscesses were idiopathic (*n* = 36, 42.4%), followed by superinfected tumors (*n* = 11, 12.9%), obstructive etiology (*n* = 10, 11.8%), and immunosuppression (*n* = 10, 11.8%). Of the tumors examined, five were adenolymphomas, four parotid cysts, one was a congenital epidermoid cyst, and one squamous cell carcinoma of unknown origin. A dental focus was present in five patients (5.9%). Two PAs were caused by a marantic parotitis and one was posttraumatic (skin lesion). Patients with a dental focus had a significantly longer hospital stay compared to patients with other etiologies (mean of 12.8 vs. 7.0 days; *p* = 0.007) (Table 4). Among the cases studied, 12 (14.1%) were classified as immunocompromised, based on diagnoses of diabetes mellitus, HIV infection, myelodysplastic syndrome, or immunosuppressive medication (Fig. 2). No significant correlations were detected between immunosuppression and the duration of antibiotics, length of hospital stay, complication rate, recurrence, or the need for parotidectomy.

**Table 4:** Statistical association between etiological variables and outcome variables

**Variable**	**Dental focus** (*n* = 5)	**No dental focus** (*n* = 80)	***p***
**Duration of antibiotics** in days, mean ± SD	12.40 ± 4.98	11.11 ± 4.68	0.556
**Hospital stay** in days, mean ± SD	12.80 ± 7.66	6.99 ± 4.37	**0.007**
**Drain duration** in days, mean ± SD	2.60 ± 0.89	3.35 ± 1.84	0.187
**Complications** n (%)	1 (20.0)	8 (10.0)	0.481
**Recurrence** n (%)	0 (0.0)	4 (5.5)	0.631
	**Chronic parotitis** (*n* = 7)	**No chronic parotitis** (*n* = 78)	***p***
**Duration of antibiotics** in days, mean ± SD	12.86 ± 8.41	11.03 ± 4.19	0.590
**Hospital stay** in days, mean ± SD	10.57 ± 9.86	7.04 ± 4.01	0.382
**Drain duration** in days, mean ± SD	5.17 ± 2.99	3.06 ± 1.48	0.147
**Complications** n (%)	2 (28.6)	7 (9.0)	0.106
**Recurrence** n (%)	1 (25.0)	3 (4.1)	0.067

*SD* Standard deviation, significance level < 0.05, significant results in bold letters

### Microbiological analysis and antibiotics

The majority of patients (*n* = 58; 68.2%) received an aminopenicillin ± beta-lactamase inhibitor as empiric antibiotic therapy, followed by cefuroxime *(n =* 13; 15.3%). The mean overall duration of antibiotic therapy (intravenous + oral administration) was 11.2 days, ranging from four to a maximum of 30 days. At one clinical site the duration of antibiotics was significantly longer (9.8 ± 3.9 days vs. 12.4 ± 4.9 days, *p* = 0.014). In 4 cases no microbiological specimen was taken. In 18 (21.2%) patients the swabs were sterile. No antibiogram was performed in specimen containing normal oral bacterial flora only. In the remaining 45 patients, antibiotic resistance testing was available and in 38 (84.4%) cases, the respective bacteria were susceptible to the empirically used antibiotic agent (Table 5). In 6 cases, the initial antibiotic therapy (Cefuroxime (2x), Aminopenicillin + beta-lactamase inhibitor (2x), Clindamycin (1x), Ciprofloxacin (1x)) was changed according to the antibiogram while in 1 patient the aminopenicillin had to be changed due to allergic reaction. Among the identified pathogens, 51 were classified as “highly relevant”, 27 as “possibly relevant” and 11 as “probably not relevant”. The most frequently detected pathogens were *Streptococci* of the *anginosus* group (*n* = 22), followed by *Staphylococcus aureus* (*n* = 6) including one case of methicillin-resistant *Staphylococcus aureus* (MRSA). One parotid abscess contained *Francisella tularensis*, a zoonotic pathogen typically found in wild animals. Its presence was most likely attributed to a prior car accident with involvement of a wild animal resulting in a skin laceration. Among the species with “possibly relevant” pathogenicity, coagulase-negative *Staphylococci* (*n *= 10) and *Cutibacterium acnes* (*n* = 7) were the most commonly detected. Additionally, four cases of different *Candida* species were identified, including one case of *Candida glabrata* (Table 6). In terms of immune status, the most frequent pathogens among immunocompetent patients were bacteria of the *Streptococcus anginosus grp.* (*n = *20, 27.4%), followed by *Staphylococcus aureus* (*n *= 4, 5.5%), *Parvimonas micra* (*n* = 4, 5.5%), and *Eikenella corrodens* (n = 4, 5.5%). Among immunocompromised patients, the most frequent pathogens were bacteria of the *Streptococcus anginosus grp.* (*n* = 2, 16.7%) and *Staphylococcus aureus* (*n* = 2, 16.7%), followed by *Klebsiella pneumoniae* (*n* = 1, 8.3%), and *Aggregatibacter aphrophilus* (*n* = 1, 8.3%). Thus, the frequency of bacteria of the *Streptococcus anginosus grp.* was higher among immunocompetent patients, whereas *Staphylococcus aureus* was more often detected among immunocompromised patients.

**Table 5:** Antibiotic therapy

Antibiotics used	*n* (%) or mean ± SD
Aminopenicillin ± beta-lactamase inhibitor	58 (68.2)
Aminopenicillin + Metronidazol	1 (1.2)
Cefuroxime	6 (7.1)
Cefuroxime + Metronidazol	7 (8.2)
Ceftriaxon	1 (1.2)
Piperacillin/Tazobactam	1 (1.2)
Clindamycin	6 (7.1)
Ciprofloxacin	1 (1.2)
Unknown	4 (4.7)
**Drain duration (days)**	9.8 ± 3.9
**According to antibiogram (*n*** **= 45)**	39 (86.7)

*SD* Standard deviation

**Table 6:** Pathogens

Pathogenicity	Germ	*n*
**Highly relevant** ***n*** **= 51**	*Streptococcus anginosus grp*	22
	*Staphylococcus aureus (MRSA)*	6 (1)
	*Parvimonas micra*	4
	*Eikenella corrodens*	4
	*Prevotella spp.*	3
	*Haemophilus influenzae*	2
	*Aggregatibacter aphrophilus*	2
	*Bacteroides spp.*	2
	*Klebsiella pneumoniae*	1
	*Pseudomonas aeruginosa*	1
	*Francisella tularensis*	1
	*Streptococcus pyogenes*	1
	*Candida glabrata*^a^	1
	*Raoultella ornithinolytica*	1
**Possibly relevant** ***n*** **= 27**	*Coagulase-negative Staphylococci*	10
	*Cutibacterium acnes*	7
	*Candida spp.*^a^	3
	*Haemophilus parainfluenzae*	2
	*Actinomyces spp.*	2
	*Corynebacterium kroppenstedtii*	1
	*Peptostreptococcus spp.*	1
	*Streptococcus pluranimalum*	1
**Probably not relevant** ***n*** **= 11**	*Oral flora only, not specified*	7
	*Streptococcus oralis*	2
	*Lactobacillus rhamnosus*	1
	*Capnocytophaga spp.*	1

^a^: C. glabrata was considered highly relevant, C. kefyr and C. tropicalis possibly relevant

*grp. group, spp. species, MRSA Methicillin-resistant staphylococcus aureus*

### Characteristics of adults and children with PA

All children (*n* = 7) and half of the adult patients with PA were treated under general anaesthesia (*p* = 0.011) (Table 7). Further, a tumorous lesion was identified as etiology in 42.9% (*n* = 3) of children and only in 10.3% (*n* = 10) of adults with PA (*p* = 0.014). No significant differences in terms of complications/recurrence were found when comparing children and adults with PA. Regarding the microbiological spectrum, we found that the most frequent pathogens in adults were bacteria of the *Streptococcus anginosus grp.* (*n* = 21, 26.9%), followed by *Staphylococcus aureus* (*n* = 5, 6.4%), *Parvimonas micra* (*n* = 4, 5.1%), and *Eikenella corrodens* (*n* = 4, 5.1%). Among children, causative pathogens were bacteria of the *Streptococcus anginosus grp*. (*n* = 1, 14.3%), *Staphylococcus aureus* (*n* = 1, 14.3%), and *Haemophilus influenzae* (*n* = 1, 14.3%).

**Table 7:** Procedural characteristics, etiology, complications/recurrence in adults versus children with PA

**Variable**	**Adults (≥ 18 years)** (*n =* 78)	**Children (< 18 years)** (*n* = 7)	***p***
**Duration of antibiotics** in days, mean ± SD	11.18 ± 4.21	11.43 ± 8.46	0.893
**Hospital stay** in days, mean ± SD	7.51 ± 4.89	5.29 ± 0.81	0.238
**Drain duration** in days, mean ± SD	3.30 ± 1.84	3.00 ± 0.82	0.743
**General anaesthesia** *n* (%)	39 (50.0)	7 (100.0)	**0.011**
**Tumor** *n* (%)	8 (10.3)	3 (42.9)	**0.014**
**Sialolithiasis** *n* (%)	10 (12.8)	0 (0.0)	0.313
**Dental Focus** *n* (%)	5 (6.4)	0 (0.0)	0.490
**Idiopathic** *n* (%)	33 (42.3)	3 (42.9)	0.978
**Chronic Parotitis** *n* (%)	6 (7.7)	1 (14.3)	0.543
**Sjögren’s** *n* (%)	2 (2.6)	0 (0.0)	0.668
**Marantic** *n* (%)	2 (2.6)	0 (0.0)	0.668
**Immunosuppression** *n *(%)	12 (15.4)	0 (0.0)	0.263
**Complications** *n* (%)	10 (12.8)	1 (14.3)	0.912
**Recurrence** *n* (%)	3 (3.8)	1 (14.3)	0.212

significance level < 0.05, significant results in bold letters

## Discussion

This present study displays the largest detailed investigation of etiology, microbiological spectrum, and surgical as well as anti-infective treatment strategies of PA, addressing a clinical challenge in head and neck surgery.

In terms of demographics, our study showed a similar sex distribution with a male predominance compared to prior literature [10]. However, the age distribution slightly differed, with the mean age in this study being 57.5 years compared to the 4th or 5th decade reported by Saibene et al. [10]. The results affirm that PA can arise across all age groups.

Overall, 14.1% of all patients in our study displayed impairment of the immune system. These findings align with prior research, which showed that immunocompromising conditions were a predisposing factor for parotid abscess [11–13]. Furthermore, oro-dental issues [14], autoimmune disorders [15], ductal and/or parenchymal disorders [12, 13, 16], superinfected adenolymphoma [17], specific disseminated diseases including melioidosis and tuberculosis [18, 19], and immunodeficiency due to conditions like HIV infection or diabetes mellitus [16, 18, 20] have been described as predisposing factors. Interestingly, although immunosuppression seems to predispose to PA, it did not have an impact on clinical course or complication rate in the present study.

Previous literature, including the review by Saibene et al., lacked detailed information on various etiologies [10]. In contrast, our study extensively investigated the etiology of parotid abscesses, offering comprehensive results that include the frequency of tumors as a contributing factor and showing that patients with a dental focus of PA had a significantly longer hospital stay than those without for the first time. The latter may be due to a lack of awareness of a dental focus as etiology of a PA despite the close anatomical relationship between the teeth of the upper jaw and the parotid gland, and consequently a delayed dental consultation. Therefore, the present data support routinely ruling out a dental focus in case of first diagnosis of PA. Further, a PA was significantly more frequently (*p* = 0.014) caused by an underlying tumorous lesion in children (42.9%) compared to adults (*p* = 0.014). Therefore, it seems that obtaining a histological specimen and applying a particularly strict follow-up in children with PA is indicated in order to exclude a possibly congenital parotid tumor as etiology of PA. Although the etiology of a high number of PA was classified as idiopathic in this study, it must be mentioned that ascending infection via Stensen’s duct or bacteremia were assumed to be the main pathomechanisms leading to PA in the previous literature [2]. The exact pathomechanism leading to PA in the cases classified as idiopathic within this study cannot be determined retrospectively.

Therapeutic management strategies involve both the drainage of pus and the administration of an appropriate antibiotic therapy [8, 11, 13, 14, 21]. Our study found abscess incision and drainage to be the most common treatment strategies, followed by aspiration either with or without radiological guidance, according to prior literature [10]. There is no study investigating differences in the type of anesthesia performed in PA. In this study a relevant number of patients underwent surgery under local anesthesia and there was no significant correlation between type of anesthesia and length of hospital stay or complication rate. Notably, neither patients treated under general, nor patients treated under local anesthesia showed postoperative impairment of the facial nerve. These results imply that both, general and local anesthesia, can be applied safely and effectively for surgical treatment of PA. Insertion of a wound drain was significantly associated with a longer hospital stay in the present study. On the other hand, patients without a drain did not show a higher rate of recurrences, more complications or a longer period of antibiotic administration compared to those with intraoperative drain insertion. However, it has to be mentioned that only 2 out of 79 surgically treated patients did not receive a drain. Therefore, the results are not sufficient to answer the question of the necessity of wound drain insertion in the surgical treatment of PA.

At one clinical site the duration of antibiotics was significantly longer (9.8 ± 3.9 days vs. 12.4 ± 4.9 days), which can be explained by a longer average hospital stay at one site compared to the other (6.2 ± 2.9 days vs. 8.4 ± 5.8 days). The most likely reason for this divergence is a more conservative intravenous antibiotic treatment duration at one site compared to the other. This reflects the missing standard of care in the duration of antibiotic treatment of PA. Prospective studies are needed to determine the ideal duration of antibiotic treatment in this disease.

Furthermore, we demonstrated a mean hospital stay of 7.3 ± 4.7 days, which was shorter than previously described in literature (13.8 ± 9.1) [11]. The shorter mean hospital stay seems justifiably against the background of a low recurrence rate of only 5.2% in the present series. Nevertheless, the length of hospital stays and the length of administration of intravenous antibiotics have to be determined depending on clinical and laboratory findings in each individual case.

In literature, antibiotic therapy typically involves aminopenicillin alone or in combination with a beta-lactamase inhibitor or clindamycin combined with third or fourth generation cephalosporins for patients with a high incidence of gram-negative bacterial infections [4, 10–14]. This is consistent with our findings, where most patients received an aminopenicillin alone or in combination with a beta-lactamase inhibitor as empiric antibiotic therapy. The mean antibiotic therapy duration was 11.2 days, consistent with prior literature recommending 10–14 days [5].

In our study, the most frequent postoperative complications were salivary fistulas agreeing with literature findings [9]. Our study revealed no new cases of facial nerve palsy during and after treatment, which was one major, but rare complication described in literature [3, 9, 10]. Furthermore, previous studies reported wound healing problems, which were not observed in our study [10]. Severe complications such as jaw osteomyelitis, temporal lobe abscess [22] craniocervical necrotizing fasciitis [23], descending mediastinitis [24], airway obstruction, septicemia, and septic shock [12, 24, 25] did not occur during the follow-up of our patients.

Prior microbiological profiling of PA revealed common pathogens like *Staphylococcus* spp., anaerobic bacteria, and *Streptococcus* spp. [4, 11, 12, 21], consistent with our findings. Furthermore, *Klebsiella* spp., *Haemophilus* spp., *Enterobacter* spp., *Prevotella* spp., *Parvimonas* spp., *Burkholderi*a spp., *Pseudomonas* spp., *Mycobacterium* spp., non-*Mycobacterium* spp., *Propionibacterium* spp., and *Brucella* spp. have also been isolated in reported cases of PA [4, 6–8, 12, 13, 15, 16, 19, 26]. In addition to the previously mentioned pathogens, our study identified *Aggregatibacter aphrophilus*, *Corynebacterium kroppenstedtii*, various *Candid*a species, a single instance of methicillin-resistant *Staphylococcus aureus* (MRSA), and *Francisella tularensis* – a zoonotic pathogen typically associated with wild animals. It is noteworthy that these pathogens have not been previously detected in cases of parotid abscess according to our knowledge. The high number of specimens containing no microorganisms is consistent with prior findings. One study found 14 of 25 cases of PA with negative bacterial cultures [5]. Prior antibiotic treatment may be the reason for the high number of specimens without detection of microorganisms. Therefore, starting systemic antibiotics after collection of the microbiological specimen may be beneficial to achieve a higher detection rate. Additionally, microbiological analysis of tissue samples instead of swabs could potentially improve the detection rate as well.

We evaluated antibiograms of the examined patients. Among cases where an antibiogram was generated, the empirically selected antibiotic agent aligned with the antibiogram results predominantly. However, in seven instances, the antibiotic therapy had to be modified following the antibiogram, which emphasizes the need of intraoperatively obtaining a microbiological specimen in order to gain precise knowledge about local species distribution and antibiotic resistances in PA. This is of special relevance since in one patient MRSA was isolated.

Moreover, the histopathologic specimen taken during surgical therapy of PA revealed a superinfected neoplasm in ten cases (five adenolymphomas, four parotid cysts, and one squamous cell carcinoma). We therefore strongly advocate considering an infected neoplasm as a differential diagnosis for PA and to routinely obtain a histological specimen in PA in order to exclude a tumorous etiology.

A limitation of this study is the retrospective study design. Additionally, factors as abscess size and the patient’s clinical condition were not considered, which could have influenced the decision of surgical technique and to opt for drainage. Moreover, we were unable to retrospectively ascertain whether antibiotic therapy had been initiated prior to microbiological examination. Further, this study exclusively contained patients with surgically treated PA. Consequently, patients with small PA which have not been captured by ultrasound imaging (i.e., in the deep parotid lobe) and have improved with medical therapy alone are not included. It therefore stays unclear if empirical medical treatment alone may be sufficient for small PA. Despite having a significantly larger case count than previous investigations, the collective size still may not adequately address rarer complications and etiologies, warranting further research in this domain. Larger multicenter studies are recommended for future research to validate and expand upon these results.

To sum up, the present study presents data on the largest described series of patients treated for PA including a comprehensive analysis of microbiological and clinical data. The study confirms that antibiotic agents and drainage are the two crucial pillars of treatment for PA being sufficient to avoid major complications. The results support the safety of surgical therapy of PA under local anesthesia. A dental examination should routinely be performed in case of PA to rule out a dental focus. Obtaining a microbiological specimen in order to modify antibiotic therapy if necessary and obtaining a histopathological specimen to rule out a tumorous etiology seems obligate.

## Data Availability

No datasets were generated or analysed during the current study.
